# Supplementary material for: Heat adaptation of phage T7 under an extended genetic code
Source: Virus Evol. 2021 Dec 1;7(2):veab100. doi: 10.1093/ve/veab100 (PMC8923235; doi:10.1093/ve/veab100)
Supplement: veab100_Supp [file veab100_supp.zip › Sequencing_Analysis.zip!/Sequencing_Analysis/S2/evidence/SNP_8.html]

BRESEQ :: Evidence


| Predicted mutation | | | | | | |
| --- | --- | --- | --- | --- | --- | --- |
| evidence | seq id | position | mutation | annotation | gene | description |
| RA | NC\_001604.1 | 27,783 | G→A | intergenic (–/–) | – / – | –/– |

| Read alignment evidence... | | | | | | | | | | | |
| --- | --- | --- | --- | --- | --- | --- | --- | --- | --- | --- | --- |
|  | seq id | position | | ref | new | freq | score (cons/poly) | reads | annotation | genes | product |
| \* | NC\_001604.1 | 27,783 | 0 | G | A | 99.3% | 13605.7 / 14.7 | 5319 | intergenic (–/–) | *–/–* | –/– |
| Reads supporting (aligned to +/- strand):  **ref** base G (19/16);  **new** base A (2752/2502);  **total** (2793/2526) | | | | | | | | | | | |
| Fisher's exact test for biased strand distribution *p*-value = 8.66e-01 | | | | | | | | | | | |
| Kolmogorov-Smirnov test that lower quality scores support variant *p*-value = 1.00e+00 | | | | | | | | | | | |

Only 100 of 5322 total aligned reads displayed.  

`GTGCTGGTTCGTTACGAGCGACCAAGTGTGGCGACTTAGTGGAAAGGCTAAGCGAAAGTTCCGTAAGTTAATCATGGAGTATCGCGATAAGATGCTTGAGAAGTATGATACTCTTTGGAATTACGTATGGGTAGGCAATACGTCCCACATTCGTTTCCTCAAGACTATCGGTGCGGTATTCCATGAAGAGTACACACGAGATGGTCAATTTCAGTTATTTACAATCACGAAAGGAGGATAACCATATGTGTTGGGCAGCCGCAATACCTATCGCTATATCTGGCGCTCAGGCTATCAGTGGTCAGAACGCTCAGGCCAAAATGATTGCCGCTCAGACCGCTGCTGGTCGTCGTCAAGCTATGGAAATCATGAGGCAGACGAACATCCAGAATGCTGACCTATCGTTGCAAGCTCGAAGTAAACTTGAGGAAGCGTCCGCCGAGTTGACCTCACAGAACATGCAGAAGGTCCAAGCTATTGGGTCTATCCGAGCGGCTATCGGAGAGAGTATGCTTGAAGGTTCCTCAATGGACCGCATTAAGCGAGTCACAGAAGGACAGTTCATTCGGGAAGCCAATATGGTAACTGAGAACTATCG  >  NC_001604.1/27483‑28080`  

                                                                                                                                                                                                                                                                                                            `|`                                                                                                                                                                                                                                                                                                           
          `gTTACGAGCGACCAAGTGTGGCGACTTAGTGGAAAGGCTAAGCGAAAGTTCCGTAAGTTAATCATGGAGTATCGCGATAAGATGCTTGAGAAGTATGATACTCTTTGGAATTACGTATGGGTAGGCAATACGTCCCACATTCGTTTCCTCAAGACTATCTGTGCGGTATTCCATGAAGAGTACACACGAGATGGTCAATTTCAGTTATTTACAATCACGAAAGGAGGATAACCATATGTGTTGGGCAGCCGCAATACCTATCGCTATATCTGGCGCTCAGGCTATCAGTGATCAGAACGCt                                                                                                                                                                                                                                                                                                 >  1:468205/1‑301 (MQ=255)`  
                `cgccaCCAAGTGTGGCGACTTGGTGGAAAGGCTAAGCGAAAGTTCCGTAAATTAATCATGGCGTCTCGCCATAAGATGCTTTAGAAGTATGTTACTCGTTGGAATTACGTATGGGTAGGCAATACGGCCCACATTCGTTTCCTCAAGACTATCGGTGCGGTATTCCATGAATATTACTCACGAGATGGTCAATTTCAGTTATTTACAATCACGAAAGGAGGATAACCATATGTGTTGGGCAGCCGCAATACCTATCGCTATATCTGGCTCTCAGGCTATCAGTGATCAGAACGCTCAGGcc                                                                                                                                                                                                                                                                                           <  1:699811/297‑1 (MQ=255)`  
                        `aGTGTGGCGACTTAGTGGAAAGGCTAAGCGAAAGTTCCGTAAGTTAATCATGGAGTATCGCGATAAGATGCTTGAGAAGTATGATACTCTTTGGAATTACGTATGGGTAGGCAATACGTCCCACATTCGTTTCCTCAAGACTATCGGTGCGGTATTCCATGAAGAGTACACACGAGATGGTCAATTTCAGTTATTTACAATCACGAAAGGAGGATAACCATATGTGTTGGGCAGCCGCAATACCTATCGCTATATCTGGCGCTCAGGCTATCAGTGATCAGAACGCTCAGGCCAAAATGAt                                                                                                                                                                                                                                                                                   >  1:284636/1‑301 (MQ=255)`  
                         `gtgtGGCGACTTAGTGGAAAGGCTAAGCGAAAGTTCCGTAAGTTAATCATGGAGTATCGCGATAAGATGCTTGAGAAGTATGATACTCTTTGGAATTACGTATGGGTAGGCAATACGTCCCACATTCGTTTCCTCAAGACTATCGGTGCGGTATTCCATGAAGAGTACACACGAGATGGTCAATTTCAGTTATTTACAATCACGAAAGGAGGATAACCATATGTGTTGGGCAGCCGCAATACCTATCGCTATATCTGGCGCTCAGGCTATCAGTGATCAGAACGCTCAGGCCAAAATGAtt                                                                                                                                                                                                                                                                                  >  1:361807/1‑301 (MQ=255)`  
                            `gggCGACTTAGTGGAAAGGCTAAGCGAAAGTTCCGTAAGTTAATCATGGAGTATCGCGATAAGATGCTTGAGAAGTATGATACTCTTTGGAATTACGTATGGGTAGGCAATACGTCCCACATTCGTTTCCTCAAGACTATCGGTGCGGTATTCCATGAAGAGTACACACGAGATGGTCAATTTCAGTTATTTACAATCACGAAAGGAGGATAACCATATGTGTTGGGCAGCCGCAATACCTATCGCTATATCTGGCGCTCAGGCTATCAGTGATCAGAACGCTCAGGCCAAAATGATTGcc                                                                                                                                                                                                                                                                               <  1:100278/300‑1 (MQ=255)`  
                                      `gTGGAAAGGCTAAGCGAAAGTTCCGTAAGTTAATCATGGAGTATCGCGATAAGATGCTTGAGAAGTATGATACTCTTTGGAATTACGTATGGGTAGGCAATACGTCCCACATTCGTTTCCTCAAGACTATCGGTGCGATATTCCATGAAGAGTACACACGAGATGGTCAATTTCAGTTATTTACAATCACGAAAGGAGGATAACCATATGTGTTGGGCAGCCGCAATACCTATCGCTATATCTGGCGCTCAGGCTATCAGTGATCAGAACGCTCAGGCCAAAATGATTGCCGCTCAGACCg                                                                                                                                                                                                                                                                     >  1:335822/1‑301 (MQ=255)`  
                                             `ggCTAAGCGAAAGTTCCGTAAGTTAATCATGGAGTATCGCGATAAGATGCTTGAGAAGTATGATACTCTTTGGAATTACGTATGGGTAGGCAATACGTCCCACATTCGTTTCCTCAAGACTATCGGTGCGGTATTCCATGAAGAGTACACACGAGATAGTCAATTTCAGTTATTTACAATCACGAAAGGAGGATAACCATATGTGTTGGGCAGCCGCAATACCTATCGCTATATCTGGCGCTCAGGCTATCAGTGATCAGAACGCTCAGGCCAAAATGATTGCCGCTCAGACCGCTGCTgg                                                                                                                                                                                                                                                              >  1:204232/1‑301 (MQ=255)`  
                                                         `gTTCCGTAAGTTACTCATGGAGTATCGCGATAAGATGCTTGAGAAGTATGATACTCTTTGGAATTACGTATGGGTAGGCAATACGTCCCACATTCGTTTCCTCAAGACTATCGGTGCGGTATTCCATGAAGAGTACACACGAGATGGTCAATTTCAGTTATTTACAATCACGAAAGGAGGATAACCATATGTGTTGGGCAGCCGCAATACCTATCGCTATATCTGGCGCTCAGGCTATCAGTGATCAGAACGCTCAGGCCAAAATGATTGCCGCTCACACCGCTGCTGGTCGTCGTCAAGc                                                                                                                                                                                                                                                  >  1:818298/1‑301 (MQ=255)`  
                                                                      `agccaggAGTATCGCGATAAGATGCTTGAAAAGTAGGATCCTCTTTGGAATTACGGATGGGTAGGCAATACGTCCCACATTCGTTTCCTCAAGACTATCGGTGCGGTATTCCATGAAGAGTACACACGAGATGGTCAATTTCAGTTATTTACAATCACGAAAGGAGGATAACCATATGTGTTGGGCAGCCGCAATACCTATCGCTATATCTGGCGCTCAGGATATCAGTGATCAGAACGCTAAGGCCAAAATGATTGCCGCTCAGACCGCTGCTGGTCGTCGTCAAGCTATGGAAATCATg                                                                                                                                                                                                                                     <  1:551485/296‑1 (MQ=255)`  
                                                                      `aTCATGGAGTATCGCGATAAGATGCTTGAGAAGTATGATACTCTTTGGAATTACGTATGGGTAGGCAATACGTCCCACATTCGTTTCCTCAAGACTATCGGTGCGGTATTCCATGAAGAGTACACACGAGATGGTCAATTTCAGTTATTTACAATCACGAAAGGAGGATAACCATATGTGTTGGGCAGCCGCAATACCTATCGCTATATCTGGCGCTCAGGCTATCAGTGATCAGAACGCTCAGGCCAAAATGATTGCCGCTCAGACCGCTGCTGGTCGTCGTCAAGCTATGGAAATCAtt                                                                                                                                                                                                                                     >  1:536097/1‑300 (MQ=255)`  
                                                                      `aTCATGGAGTATCGCGATAAGATGCTTGAGAAGTATGATACTCTTTGGAATTACGTATGGGTAGGCAATACGTCCCACATTCGTTTCCTCAAGACTATCGGTGCGGTATTCCATGAAGAGTACACACGAGATGGTCAATTTCAGTTATTTACAATCACGAAAGGAGGATAACCATATGTGTTGGGCAGCCGCAATACCTATCGCTATATCTGGCGCTCAGGCTATCAGTGATCAGAACGCTCAGGCCAAAATGATTGCCGCTCAGACCGCTGCTGGTCGTCGTCAAGCTATGGAAATCATg                                                                                                                                                                                                                                     <  1:321468/301‑1 (MQ=255)`  
                                                                      `aTCATGGAGTATCGCGATAAGATGCTTGAGAAGTATGATACTCTTTGGAATTACGTATGGGTAGGCAATACGTCCCACATTCGTTTCCTCAAGACTATCGGTGCGGTATTCCATGAAGAGTACACACGAGATGGTCAATTTCAGTTATTTACAATCACGAAAGGAGGATAACCATATGTGTTGGGCAGCCGCAATACCTATCGCTATATCTGGCGCTCAGGCTATCAGTGATCAGAACGCTCAGGCCAAAATGATTGCCGCTCAGACCGCTGCTGGTCGTCGTCAAGCTATGGAAATCATg                                                                                                                                                                                                                                     <  1:498986/301‑1 (MQ=255)`  
                                                                              `gTATCGCGATAAGATGCTTGAGAAGTATGATACTCTTTGGAATTACGTATGGGTAGGCAATACGTCCCACATTCGTTTCCTCAAGACTATCGGTGCGGTATTCCATGAAGAGTACACACGAGATGGTCAATTTCAGTTATTTACAATCACGAAAGGAGGATAACCATATGTGTTGGGCAGCCGCAATACCTATCGCTATATCTGGCGCTCAGGCTATCAGTGATCAGAACGCTCAGGCCAAAATGATTGCCGCTCAGACCGCTGCTGGTCGTCGTCAAGCTATGGAAATCATGAGGCAGAc                                                                                                                                                                                                                             >  1:162681/1‑301 (MQ=255)`  
                                                                                `aTCGCGATAAGATGCTTGAGAAGTATGATACTCTTTGGAATTACGTATGGGTAGGCAATACGTCCCACATTCGTTTCCTCAAGACTATCGGTGCGGTATTCCATGAAGAGTACACACGAGATGGTCAATTTCAGTTATTTACAATCACGAAAGGAGGATAACCATATGTGTTGGGCAGCCGCAATACCTATCGCTATATCTGGCGCTCAGGCTATCAGTGATCAGAACGCTCAGGCCAAAATGATTGCCGCTCAGACCGCTGCTGGTCGTCGTCAAGCTATGGAAATCATGAGGCAGACGa                                                                                                                                                                                                                           <  1:148388/301‑1 (MQ=255)`  
                                                                                `aTCGCGATAAGATGCTTGAGAAGTATGATACTCTTTGGAATTACGTATGGGTAGGCAATACGTCCCACATTCGTTTCCTCAAGACTATCGGTGCGGTATTCCATGAAGAGTACACACGAGATGGTCAATTTCAGTTATTTACAATCACGAAAGGAGGATAACCATATGTGTTGGGCAGCCGCAATACCTATCGCTATATCTGGCGCTCAGGCTATCAGTGATCAGAACGCTCAGGCCAAAATGATTGCCGCTCAGACCGCTGCTGGTCGTCGTCAAGCTATGGAAATCATGAGGCAGACGa                                                                                                                                                                                                                           <  1:231993/301‑1 (MQ=255)`  
                                                                                      `aTAAGATGCTTGAGAAGTATGATACTCTTTGGAATTACGTATGGGTAGGCAATACGTCCCACATTCGTTTCCTCAAGACTATCGGTGCGGTATTCCATGAAGAGTACACACGAGATGGTCAATTTCAGTTATTTACAATCACGAAAGGAGGATAACCATATGTGTTGGGCAGCCGCAATACCTATCGCTATATCTGGCGCTCAGGATATCAGTGATCAGAACGCCCAGGCCAAAATGATTGCAGCTCAGACCGCTGCTGGTCGTCGCCAAGCTATGGAAATAATGAGGCAGACGAACATcc                                                                                                                                                                                                                     >  1:171192/1‑301 (MQ=255)`  
                                                                                         `aGATGCTTGAGAAGTATGATACTCTTTGGAATTACGTATGGGTATGCAATACGTCCCACATTCGTTTCCTCAAGACTATCGGTGCGGTATTCCATGAAGAGTACACACGATATGGTCAATTTCAGTTATTTACAATCACGAAAGGAGGATAACCATATGTGTTGGGCAGCCGCAATACCTATCGCTATATCTGGCGCTCAGGCTATCAGTGATCAGAACGCTCAGGCCAAAATGATTGCCGCTCAGACCGCTGCTGGTCGTCGTCAAGCTATGGAAATCATGAGGCAGACGAACATCCAGa                                                                                                                                                                                                                  <  1:114056/301‑1 (MQ=255)`  
                                                                                          `gATGCTTGAGAAGTATGATAATCTTGGGAATTACGTATGGGTAGGCAATACGTCCCACATTCGTTTCCTCAAGACTATCGGTGCGGTAGTCCATGAAGAGTACACACGAGATGGTCAATTTCAGTTATTTACAATCACGAAAGGAGGATAACCATATGTGTTGGGCAGCCGCAATACCTATCGCTATATCTGGCGCTCAGGCTATCAGTGATCAGAACGCTCAGGCCAAAATGATTGCCGCTCAGACCGCCTCTGGTCGTCGTCAAGCTATGGAAATCATGAGGCAGACGAACATCCAGaa                                                                                                                                                                                                                 <  1:665763/301‑1 (MQ=255)`  
                                                                                             `gCTTGAGAAGTATGATACTCTTTGGAATTACGTATGGGTAGGCAATACGTCCCACATTCGTTTCCTCAAGACTATCGGTGCGGTATTCCATGAAGAGTACACACGAGATGGTCAATTTCAGTTATTTACAATCACGAAAGGAGGATAACCATATGTGTTGGGCAGACGCAATACCTATCGCTATATCTGGCGCTCAGGCTATCAGTGATCAGAACGCTCAGGCCAAAATGATTGCCGCTCAGACCGCTGCTGGTCGTCGTCAAGCTATGGAAATCATGAGGCAGACGAACATCCAGAATGc                                                                                                                                                                                                              >  1:484987/1‑301 (MQ=255)`  
                                                                                              `cTTGAGAAGTATGATACTCTTTGGAATTACGTATGGGTAGGCAATACGTCCCACATTCGTTTCCTCAAGACTATCGGTGCGGTATTCCATGAAGAGTACACACGAGATGGTCAATTTCAGTTATTTACAATCACGAAAGGAGGATAACCATATGTGTTGGGCAGCCGCAATACCTATCGCTATATCTGGCGCTCAGGCTATCAGTGATCAGAACGCTCAGGCCAAAATGATTGCCGCTCAGACCGCTGCTGGTCGTCGTCAAGCTATGGAAATCATGAGGCAGACGAACATCCAGAATGCt                                                                                                                                                                                                             >  1:558921/1‑301 (MQ=255)`  
                                                                                              `cTTGAGAAGTATGATACTCTTTGGAATTACGTATGGGTAGGCAATACGTCCCACATTCGTTTCCTCAAGACTATCGGTGCGGTATTCCATGAAGAGTACACACGAGATGGTCAATTTCAGTTATTTACAATCACGAAAGGAGGATAACCATATGTGTTGGGCAGCCGCAATACCTATCGCTATATCTGGCGCTCAGGCTATCAGTGATCAGAACGCTCAGGCCAAAATGATTGCCGCTCAGACCGCTGCTGGTCGTCGTCAAGCTATGGAAATCATGAGGCAGACGAACATCCAGAATGCt                                                                                                                                                                                                             >  1:824874/1‑301 (MQ=255)`  
                                                                                                   `gaAGTATGATACTCTTTGGAATTACGTATGGGTAGGCAATACGTCCCACATTCGTTTCCTAAAGACTATCGGTGCGGTATTCCATGAAGAGTACACACGAGATGGTCAATTTCAGTTATTTACAATCACGAAAGGAGGATAACCATATGTGTTGGGCAGCCGCAATACCTATCGCTATATCTGGCGCTCAGGCTATCAGTGATCAGAAAGCTCAGGCCAAAATGATTGCCGCTCAGACCGCTGCTGGTCGTCGTAAAGCTATGGAAATCATGAGGCAGACGAACATCCAGAATGCTGaaca                                                                                                                                                                                                        >  1:611653/1‑298 (MQ=255)`  
                                                                                                      `gTATGATACTCTTTGGAATTACGTATGGTTAGGCAATACGTCCCACATTCGTTTCCTCAAGACTATCGGTTCGGTATTCCATGAAGAGTACACACGAGATGGTCAATTTCAGTTATTTACAATCACGAAAGGAGGATAACCATATGTGTTGGGCAGCCGCAATACCTATCGCTATATCTGGCGCTCAGGCTATCAGTGATCAGAACGCTCAGGCCAACATGATTGCCGCTCATACCGCTGCTGGTCGTCGTCAAGCTATGGAAATCATTAGGCAGACGAACATCCATAATGCTTACCTacc                                                                                                                                                                                                     >  1:493113/1‑299 (MQ=255)`  
                                                                                                      `gTATGATACTCTTTGGAATTACGTATGGGTAGGCAATACGTCCCACATTCGTTTCCTCAAGACTATCGGTGCGGTATTCCATGAAGAGTACACACGAGATTGTCAATTTCAGTTATTTACAATCACGAAAGGAGGATAACCATATGTGTTGGGCAGCCGCAATACCTATCGCTATAACTGGCGCTCAGGCTATCAGTGATCAGAACGCTCAGGCCAAAATGCTTGCCGCTCAGACCGCTGCGGGTCGTCGTCAAGCTATGGAAATCATGAGGCAGACGAACATCCAGAATGCTGACCAAtg                                                                                                                                                                                                     >  1:634755/1‑300 (MQ=255)`  
                                                                                                      `gTATGATACTCTTTGGAATTACGTATGGGTAGGCAATACGTCCCACATTCGTTTCCTCAAGACTATCGGTGCGGTATTCCATGAAGAGTACACACGAGATGGTCAATTTCAGTTATTTACAATCACGAAAGGAGGATAACCATATGTGTTGGGCAGCCGCAATACCTATCGCTATATCTGGCGCTCAGGCTATCAGTGATCAGAACGCTCAGGCCAAAATGATTGCCGCTCAGACCGCTGCTGGTCGTCGTCAAGCTATGGAAATCATGAGGCAGACGAACATCCAGaactgtctcttata                                                                                                                                                                                                     >  1:783920/1‑289 (MQ=255)`  
                                                                                                      `gTATGATACTCTTTGGAATTACGTATGGGTAGGCAATACGTCCCACATTCGTTTCCTCAAGACTATCGGTGCGGTATTCCATGAAGAGTACACACGAGATGGTCAATTTCAGTTATTTACAATCACGAAAGGAGGATAACCATATGTGTTGGGCAGCCGCAATACCTATCGCTATATCTGGCGCTCAGGCTATCAGTGATCAGAACGCTCAGGCCAAAATGATTGCCGCTCAGACCGCTGCTGGTCGTCGTCAAGCTATGGAAATCATGAGGCAGACGAACATCCAGAATGCTGACCTATc                                                                                                                                                                                                     >  1:277555/1‑301 (MQ=255)`  
                                                                                                      `gTATGATACTCTTTGGAATTACGTATGGGTAGGCAATACGTACCACATTCGTTTCCTCAAGACTATCGGTGCGGTATTCAATGAAGAGTACACACGAGATGGTCAATTTCAGTTATTTACAATCACGAAAGGAGGATAACCATATGTGTTGGGCAGCCGCAATACCTATCGCTATATCTGGCGCTCAGGCTATCAGTGATCAGAACGCTCAGGCCAAAATGATTGCCGCTCAGACCGCTGCTGGTCGTCGTCAAGCTATGGAAATCATGAGGCAGACGAACATCCAGAATGCTGAccctgt                                                                                                                                                                                                     >  1:458830/1‑297 (MQ=255)`  
                                                                                                                `ctTTGGAATTACGTATGGGTAGGCAATACGTCCCACATTCGTTTCCTCAAGACTATCGGTGCGGTATTCCATGAAGAGTACACACTAGATGGTCAATTTCAGTTATTTACAATCACGAAAGGAGGATAACCATATGTGTTGGGCAGCCGCAATACCTATCGCTATATCTGGCGCTCAGGCTATCAGTGATCAGAACGCTCAGGCCAAAATGATTGCCGCTCAGACCGCTGCTGGTCGTCGTCAAGCTATGGAAATCATGAGGCAGACGAACATCCAGAATGCTGACCTATCGTTGCactgt                                                                                                                                                                                           >  1:357125/1‑297 (MQ=255)`  
                                                                                                                     `gAATTACGTATGGGTAGGCAATACGTCCCACATTCGTTTCCTCAAGACTATCGGTGCGGTATTCCATGAAGAGTACACACGAGATGGTCAATTTCAGTTATTTACAATCACGAAAGGAGGATAACCATATGTGTTGGGCAGCCGCAATACCTATCGCTATATCTGGCGCTCAGGCTATCTGTGATCAGAACGCTCAGGCCAAAATGATTGCCGCTCAGACCGCTGCTGGTCGTCGTCAAGCTATGGAAATCATGAGGCAGACGAACATCCAGAATGCTGACCTATCGTTGCAAGCTCGAAg                                                                                                                                                                                      <  1:712989/301‑1 (MQ=255)`  
                                                                                                                     `gAATTACGTATGGGTAGGCAATACGTCCCACATTCGTTTCCTCAAGACTATCGGTGCGGTATTCCATGAAGAGTACACACGAGATGGTCAATTTCAGTTATTTACAATCACGAAAGGAGGATAACCATATGTGTTGGGCAGCCGCAATACCTATCGCTATATCTGGCGCTCAGGCTATCAGTGATCAGAACGCTCAGGCCAAAATGATTGCCGCTCAGACCGCTGCTGGTCGTCGTCAAGCTATGGAAATCATGAGGCAGACGAACATCCAGAATGCTGACCTATCGTTGCAAGCTCGAAg                                                                                                                                                                                      <  1:436515/301‑1 (MQ=255)`  
                                                                                                                          `aCGTATGGGTAGGCAATACGTCCCACATTCGTTTCCTCAAGACTATCGGTGCGGTATTCCATGAAGAGTACACACGAGATGGTCAATTTCAGTTATTTACAATCACGAAAGGAGGATAACCATATGTGTTGGGCAGCCGCAATACCTATCGCTATCTCTGTCGCCCAGGCTATCAGTGATCTGAACGCTCAGGCCAAAATGCTTGCCGCTCAGACCGCTGCTGGTCGTCTTCAAGCTATGGAAATCATTCGGCAGGCGAACATCCAGAATGCTTACCTATCGTTGCAAGCTCGATGTAAAc                                                                                                                                                                                 >  1:477654/1‑301 (MQ=255)`  
                                                                                                                          `aCGTATGGGTAGGCAATACGTCCCACATTCGTTTCCTCAAGACTATCGGTGCGGTATTCCATGAAGAGTACACACGAGATGGTCAATTTCAGTTATTTACAATCACGAAAGGAGGATAACCATATGTGTTGGGCAGCCGCAATACCTATCGCTATATCTGGCGCTCAGGCTATCAGTGATCAGAACGCTCTGGCCAAAATGATTGCCGCTCAGACCGCTGCTGGTGGTCGGCAAGCTAGGGAAATCATGAGGCAGACGAACATCCAGAATGCTGACCGCTCGTTGCAAGCTctgcctctta                                                                                                                                                                                 >  1:802626/1‑292 (MQ=255)`  
                                                                                                                          `aCGTATGGGTAGGCAATACGTCCCACATTCGTTTCCTCAAGACTATCGGTGCGGTATTCCATGAAGAGTACACACGAGATGGTCAATTTCAGTTATTTACAATCACGAAAGGAGGATAACCATATGTGTTGGGCAGCCGCAATACCTATCGCTATATCTGGCGCTCAGGCTATCAGTGATCAGAACGCTCAGGCCAAAATGATTGCCGCTCAGACCGCTGCTGGTCGTCGTCAAGCTATGGAAATCATGAGGCAGACGAACATCCAGAATGCTGACCTATCGTTGCAAGCCCGAAGGaaaa                                                                                                                                                                                 >  1:218756/1‑300 (MQ=255)`  
                                                                                                                           `cGTATGGGTAGGCAATACGTCCCACATTCGTTTCCTCAAGACTATCGGTGCGGTATTCCATGAAGAGTACACACGAGATGGTCAATTTCAGTTATTTACAATCACGAAAGGAGGATAACCATATGTGTTGGGCAGCCGCAATACCTATCGCTATATCTGGCGCTCAGGCTATCAGTGATCAGAACGCTCAGGCCAAAATGATTGCCGCTCAGACCGCTGCTGGTCGTCGTCAAGCTATGGAAATCATGAGGCAGACGAACATCCAGAATGCTGACCTATCGTTGCAAGCTCGAAGTAAACt                                                                                                                                                                                <  1:445332/301‑1 (MQ=255)`  
                                                                                                                               `tGGGTAGGCAATACGTCCCACATTCGTTTCCTCAAGACTATCGGTGCGGTATTCCATGAAGAGTACACACGAGATGGTCAATTTCAGTTATTTACAATCACGAAAGGAGGATAACCATATGTGTTGGGCAGCCGCAATACCTATCGCTATATCTGGCGCTCAGGCTATCAGTGATCAGAACGCTCAGGCCAAAATGATTGCCGCTCAGACCGCTGCTGGTCGTCGTCAAGCTATGGAAATCATGAGGCAGACGAACATCCAGAATGCTGACCTATCGTTGCAAGCTCGAAGTAAACTTGAg                                                                                                                                                                            >  1:314169/1‑301 (MQ=255)`  
                                                                                                                                   `tAGGCAATACGTCCCACATTCGTTTACTCAAGACTATCGGTGCGGGATTCCATGAAGAGTACACACGAGATGGTCAATTTCAGTTATTTACAATCACGAAAGGAGGATAACCATATGTGTTGGGCAGCCGCAATACCTATCGCTATATCTGGCGCTCAGGCTATCAGTGATCAGAACGCTCAAGCCAAAATGATTGCCGCTCAGACCGCTGCTGGTCGTCGTCAAGCTATGGAAATCATGAGGCAGACGAACATCCAGAATGCTGACCTATCGTTGCAAGCTCGAAGTAAACTTGaagaag                                                                                                                                                                        <  1:565986/301‑1 (MQ=255)`  
                                                                                                                                    `aGGCAATACGTCCCACATTCGTTTCCTCAAGACTATCGGTGCGGTATTCCATGAAGAGTACACACGAGATGGTCAATTTCAGTTATTTACAATCACGAAAGGAGGATAACCATATGTGTTGGGCAGCCGCAATACCTATCGATATATCTGGAGCTCAGGCTATCAGTGATCAGAACGCTCAGGCCAAAATGATTGCCGCTCAGACCGCTGCTGGTAGTCGTCAAGCTATGGAAATCATGAGGCAGACGAACATCCAGAATGCTGACCTATCGTTGCAAGCTCGAAGTAAACTTGAGGAAGc                                                                                                                                                                       >  1:155804/1‑301 (MQ=255)`  
                                                                                                                                     `ggCAATACGTCCCACATTCGTTTCCTCAAGACTATCGGTGCGGTATTCCATGAAGAGTACACACGAGATGGTCAATTTCAGTTATTTACAATCACGAAAGGAGGATAACCATATGTGTTGGGCAGCCGCAATACCTATCGCTATATCTGGCGCTCAGGCTATCAGTGATCAGAAAGCTCAGGCCAAAATGATTGCCGCTCAGACCGCTGCTGTTCGTCGTCAAGCTATGGAAATCATGAGGCAGACGAACATCCAGAATGCTGACCTATCGTTGAAAGCTCGAAGTAAACTTGCGGAACCg                                                                                                                                                                      >  1:514071/1‑301 (MQ=255)`  
                                                                                                                                      `gCAATACGTCCCACATTCGTCTCCTCAAGACTATCGGTGCGGTATTCCATGAAGAGTACACACGAGATGGTCAATTTCAGTTATTTACAATCACGAAAGGAGGATAACCATATGTGTTGGGCAGCCGCAATACCTATCGCTATATCTGGCGCTCAGGCTATCAGTGATCAGAACGCTCAGGCCAAAATGATTGCCGCTCAGACCGCTGCTGGTCGTCGTCACGCTATGGAAATCATGAGGCAGACGAACATCCAGAATGCTGACCTATCGTTGCAAGCTCGAAGTAAACTTGAGGAAGCGt                                                                                                                                                                     >  1:774714/1‑301 (MQ=255)`  
                                                                                                                                               `cccACATTCGTTTCCTCAAGACTATCGGTGCGGTATTCCATGAAGAGTACACACGAGATGGTCAATTTCAGTTATTTACAATCACGAAAGGAGGATAACCATATGTGTTGGGCAGCCGCAATACCTATCGCTATATCTGGTGCTCAGGCTATCAGTGATCAGAACGCTCAGGCCAAAATGATTGCCGCTCAGACCGCTGCTGGTCGTCGTCAAGCTATGGAAATCATGAGGCAGACGAACATCCAGAATGCTGACCTATCGTTGCAAGCTAGAAGTAAACTTGAGGAAGCgctgtctctta                                                                                                                                                            >  1:673388/1‑291 (MQ=255)`  
                                                                                                                                                         `tttCCTCAAGACTATCGGTGCGGTATTCCATGAAGAGTACACACGAGATGGTCAATTTCAGTTATTTACAATCACGAAAGGAGGATAACCATATGTGTTGGGCAGCCGCAATACCTATCGCTATATCTGGCGCTCAGGCTATCAGTGATCAGAACACTCAGGCCAAAATGATTGCCGCTCAGACCGCTGCTGGTCGTCGTCAAGCTATGGAAATCATGAGGCAGACGAACATCCAGAATGCTGACCTATCGTTGCAAGCTCGAAGTAAACTTGAGGAAGCGTCCTCCGAGTTGACCTcaca                                                                                                                                                  >  1:291060/1‑301 (MQ=255)`  
                                                                                                                                                            `ccTCAAGACTATCGGTGCGGTATTCCATGAAGAGTACACACGAGATGGTCAATTTCAGTTATTTACAATCACGAAAGGAGGATAACCATATGTGTTGGGCAGCCGCAATACCTATCGCTATATCTGGCGCTCAGGCTATCAGTGATCAGAACGCTCAGGCCAAAATGATTGCCGCTCAGACTGCTGCTGGTCGTCGTCAAGCTATGGAAATCATGAGGCAGACGAACATCCAGAATGCTGACCTATCGTTGCAAGCTCGAAGTAAACTTGAGGAAGCGTCCGCCGAGTTGAccctgtctct                                                                                                                                               >  1:210789/1‑293 (MQ=255)`  
                                                                                                                                                             `cTCAAGACTATCTGTGCGGTATGCAATGAAGAGTACACACGAGATGGTCAATTTCAGTTAGTTACAATCACGAAAGGAGGATAACCATATGTGTTGGGCCGCCGCAATACCTATCGCTATATCTGGCGCTCAGGCTATCAGTGATCATAACGCTCAGGCCAAAATGATTGCCGCTCAGACCGCTGCTGGTCGTCGTCAAGCTATTGAAATCATGAGGCAGACGAACATCCCGAATGCTGACCTATCGTTACAAGCTCGAAGTAAACTTGAGGAAGCGTCCGCCGAGTTGACCTCACAGacc                                                                                                                                              <  1:128365/301‑3 (MQ=255)`  
                                                                                                                                                                  `gACTATCGGTGCGGTATTCCATGAAGAGTACACACGAGATGGTCAATTTCAGTTATTTACAATCACGAAAGGAGGATAACCATATGTGTTGGGCAGCCGCAATACCTATCGCTATATCTGGCGCTCAGGCTATCAGTGATCAGAACGCTCAGGCTACAATGATTGCCGCTCAGACCGCTGCTGGTCGTCGTCAAGCTATGGAAATCATGAGGCAGACGAACATCCAGAATGCTGACCTATCGTTGCAAGCTCGAAGTAAACTTGAGGAAGCGTCCGCCGAGTTGACCTCCCAGAACATGCa                                                                                                                                         >  1:751211/1‑301 (MQ=255)`  
                                                                                                                                                                         `ggTGCGGTATTCCATGAAGAGTACACACGAGATGGTCAATTTCAGTTATTTACAATCACGAAAGGAGGATAACCATATGTGTTGGGCAGCCGCAATACCTATCGCTATATCTGGCGCTCAGGCTATCAGTGATCAGAACGCTCAGGCCAAAATGATTGCCGCTCAGACCGCTGCTGGTCGTCGTCAAGCTATGGAAATCATGAGGCAGACGAACATCCAGAATGCTGACCTATCGTTGCAAGCTCGAAGTAAACTTGAGGAAGCGTCCGCCGAGTTGACCTCACAGAACATGCAGTAGGTc                                                                                                                                  >  1:507000/1‑301 (MQ=255)`  
                                                                                                                                                                          `gTGCGGTATTCCATGAAGAGTACACACGAGATGGTCAATTTCAGTTATTTACAATCACGAAAGGAGGATAACCATATGTGTTGGGCAGCCGCAATACCTATCGCTATATCTGGCGCTCAGGCTATCAGTGATCAGAACGCTCAGGCCAAAATGATTGCCGCTCAGACCGCTGCTGGTCGTCGTCAAGCTATGGAAATCATGAGGCAGACGAACATCCAGAATGCTGACCTATCGTTGCAAGCTCGAAGTAAACTTGAGGAAGCGTCCGCCGAGTTGACCTCACAGAACATGCAGAAGGTcc                                                                                                                                 <  1:421879/301‑1 (MQ=255)`  
                                                                                                                                                                          `gTGCGGTATTCCATGAAGAGTACACACGAGATGGTCAATTTCAGTTATTTACAATCACGAAAGGAGGATAACCATATGTGTTGGGCAGCCGCAATACCTATCGCTATATCTGGCGCTCAGGCTATCAGTGATCAGAACGCTCAGGCCAAAATGATTGCCGCTCAGACCGCTGCTGGTCGTCGTCAAGCTATGGAAATCATGAGGCAGACGAACATCCAGAATGCTGACCTATCGTTGCAAGCTCGAAGTAAACTTGAGGAAGCGTCCGCCGAGTTGACCTCACAGAACATGCAGAAGGTcc                                                                                                                                 >  1:604147/1‑301 (MQ=255)`  
                                                                                                                                                                              `cagaTTCCATGAAGAGTACACACGAGATGGTCAATTTCAGTTATTTACAATCACGAAAGGAGGATAACCATATGTGTTGGGCAGCCGCAATACCTATCGCTATATCTGGCGCTCAGGCTATCAGTGATCAGAACGCTCAGGCCAAAATGATTGCCGCTCAGACCGCTGCTGGTCGTCGTCAAGCTATGGAAATCATGAGGCAGACGAACATCCAGAATGCTGACCTATCGTTGCAAGCTCGAAGTAAACTTGAGGAAGCGTCCGCCGAGTTGACCTCACAGAACATGCAGAAGGTCCAAGc                                                                                                                             <  1:375894/298‑1 (MQ=255)`  
                                                                                                                                                                               `gTATTCCATGAAGAGTACACACGAGATGGTCAATTTCAGTTATTTACAATCACGAAAGGAGGATAACCATATGTGTTGGGCAGCCGCAATACCTATCGCTATATCTGGCGCTCAGGCTATCAGTGATCAGAACGCTCAGGCCAAAATGATTGCCGCTCAGACCGCTGCTGGTCGTCGTCAAGCTATGGAAATCATGAAGCAGACGAACATCCAGAATGCTGACCTATCGTAGCAAGCTCGAAGTAAACTTGAGGAAGCGTCCGCCGAGTTGACCTCACAGAACATGCAGAAGGTCCAAGcc                                                                                                                            >  1:527223/1‑300 (MQ=255)`  
                                                                                                                                                                               `aagggacaggAAGAGTACCCACGAGATTGTCAATTTCAGTTATTTACAATCACGAAAGGAGGATAACCATATGTGTTGGGCAGCCGCAATACCTATCGCTATATCTGGCGCTCAGGCTATCAGTGATCAGAACGCTCAGGCCAAAATGATTGCCGCTCAGACCACTGCTGGTCGTCGTCAAGCTATGGAAATCATGAGGCAGACGAACATCCAGAATGCTGACCTATCGTTGCAAGCTCGAAGTAAACTTGAGGAAGCGTCCGCCGAGTTGACCTCACAGAACATGCAGAAGGTCCAAGCt                                                                                                                            <  1:414417/292‑1 (MQ=255)`  
                                                                                                                                                                                 `aTTCCATGAAGAGTACACACGAGATGGTCAATTTCAGTTATTTACAATCACGAAAGGAGGATAACCATATGTGTTGGGCAGCCGCAATACCTATCGCTATATCTGGCGCTCAGGCTATCAGTGATCAGAACGCTCAGGCCAAAATGATTGCCGCTCAGACCGCTGCTGGTCGTCGTCAAGCTATGGAAATCATGAGGCAGACGAACATCCAGAATGCTGACCTATCGTTGCAAGCTCGAAGTAAACTTGAGGAAGCGTCCGCCGAGTTGACCTCACAGAACactgtctcttatacacatct                                                                                                                          >  1:107458/1‑282 (MQ=255)`  
                                                                                                                                                                                   `tCCATGAAGAGTACACACGAGATGGTCAATTTCAGTTATTTACAATCACGAAAGGAGGATAACCATATGTGTTGGGCAGCCGCAATACCTATCGCTATATCTGGCGCTCAGGCTATCAGTGATCAGAACGCTCAGGCCAAAATGATTGCCGCTCAGACCGCTGCTGGTCGTCGTCAAGCTATGGAAATCATGAGGCAGACGAACATCCAGAATGCTGACCTATCGTTGCAAGCTCGAAGTAAACTTGAGGAAGCGTCCGCCGAGTTGACCTCACAGAACATGCAGAAGGTCCAAGCTATTg                                                                                                                        >  1:429292/1‑301 (MQ=255)`  
                                                                                                                                                                                   `tCCATGAAGAGTACACACGAGATGGTCAATTTCAGTTATTTACAATCACGAAAGGAGGATAACCATATGTGTTGGGCAGCCGCAATACCTATCGCTATATCTGGCGCTCAGGCTATCAGTGATCAGAACGCTCAGGCCAAAATGATTGCCGCTCAGACCGCTGCTGGTCGTCGTCAAGCTATGGAAATCATGAGGCAGACGAACATCCAGAATGCTGACCTATCGTTGCAAGCTCGAAGTAAACTTGAGGAAGCGTCCGCCGAGTTGACCTCACAGAACATGCAGAAGGTCCAAGCTATTg                                                                                                                        >  1:13521/1‑301 (MQ=255)`  
                                                                                                                                                                                   `tCCATGAAGAGTACACACGAGATGGTCAATTTCAGTTATTTACAATCACGAAAGGAGGATAACCATATGTGTTGGGCAGCCGCAATACCTATCGCTATATCTGGCGCTCAGGCTATCAGTGATCAGAACGCTCAGGCCAAAATGATTGCCGCTCAGACCGCTGCTGGTCGTCGTCAAGCTATGGAAATCATGAGGCAGACGAACATCCAGAATGCTGACCTATCGTTGCAAGCTCGAAGTAAACTTGAGGAAGCGTCCGCCGAGTTGACCTCACAGAACATGCAGAAGGTCCAAGCTATTg                                                                                                                        >  1:327958/1‑301 (MQ=255)`  
                                                                                                                                                                                   `tCCATGAAGAGTACACACGAGATGGTCAATTTCAGTTATTTACAATCACGAAAGGAGGATAACCATATGTGTTGGGCAGCCGCAATACCTATCGCTATATCTGGCGATCAGGCTATCAGTGATCAGAACGCTCAGGCCAAAATGATTGCCGCTCAGACCGCTGCTGGTCGTCGTCAAGCTATGGAAATCATGAGGCAGACGAACATCCAGAATGCTGACCTATCGTTGCAAGCTCGAAGTAAACTTGAGGAAGCGTCCGCCGAGTTGACCTCACAGAACATGCAGAAGGTCCAAGccctgt                                                                                                                        >  1:8573/1‑296 (MQ=255)`  
                                                                                                                                                                                    `ccATGAAGAGTACACACGAGATGGTCAATTTCAGTTATTTACAATCACGAAAGGAGGATAACCATATGTGTTGGGCAGCCGCAATACCTATCGCTATATCTGGCGCTCAGGCTATCAGTGATCAGAACGCTCAGGCCAAAATGATTGCCGCTCAGACCGCTGCTGGTCGTCGTCAAGCTATGGAAATCATGAGGCAGACGAACATCCAGAATGCTGACCTATCGTTGCAAGCTCGAAGTAAACTTGAGGAAGCGTCCGCCGAGTTGACCTCACAGAACCTgtctcttatacacatctccga                                                                                                                       >  1:726669/1‑281 (MQ=255)`  
                                                                                                                                                                                     `cATGAAGAGTACACACGAGATGGTCAATTTCAGTTATTTACAATCACGAAAGGAGGATAACCATATGTGTTGGGCAGCCGCAATACCTATCGCTATATCTGGCGCTCAGGCTATCAGTGATCAGAACGCTCAGGCCAAAATGATTGCCGCTCAGACCGCTGCTGGTCGTCGTCAAGCTATGGAAATCATGAGGCAGACGAACATCCAGAATGCTGACCTATCGTTGCAAGCTCGAAGTAAACTTGAGGAAGCGTCCGCCGAGTTGACCTCACAGAACATGCAGactgtctcttatacacat                                                                                                                      >  1:452138/1‑284 (MQ=255)`  
                                                                                                                                                                                     `cATGAAGAGTACACACGAGATGGTCAATTTCAGTTATTTACAATCACGAAAGGAGGATAACCATATGTGTTGGGCAGCCGCAATACCTATCGCTATATCTGGCGCTCAGGCTATCAGTGATCAGAACGCTCAGGCCAAAATGATTGCCGCTCAGACCGCTGCTGGTCGTCGTCAAGCTATGGAAATCATGAGGCAGACGAACATCCAGAATGCTGACCTATCGTTGCAAGCTCGAAGTAAACTTGAGGAAGCGTCCGCCGAGTTGACCTCACAGAACATGCAGAAGGTCCAAGCTATTggg                                                                                                                      <  1:261846/301‑1 (MQ=255)`  
                                                                                                                                                                                      `tcggagatgtgtataagagacagcAAGTTCAGTTATTTACAATCACGAAAGGAGGATAACCATATGTGTTGGGCAGCCGCAATACCTATCGCTATATCTGGCGCTCAGGCTATCAGTGATCAGAACGCTCAGGCCAAAATGAATGCCGCTCAGACCGCTGCTGGTCGTCGTCAAGCTATGGAAATCATGAGGCAGACGAACATCCAGAATGCTGACCTATCGTTGCAAGCTCGAAGTAAACTTGAGGAAGCGTCCGCCGAGTTGACCTCACAGAACATGCAGAAGGTCCAAGCTATTGGGt                                                                                                                     <  1:241547/278‑1 (MQ=255)`  
                                                                                                                                                                                      `aggAAAAGTACAAACGAGATGTCCAATTTCAGTAATTTACAATCACGAAAGGAGGATAACCATATGGTTTGGGCAGCCGCAATACCTACCGCTATATCTGGCGCTCAGGCTATCAGTGATCAGAACGTTCA‑GCCCAAATGATTGCCGCTCAGACCGCTGCTGGTCGTCGTCAAGCTATGGAAATCATGAGGCAGACGAACATCCAGAATGCTGACCTATCGTTGCAAGCTCGAAGTAAACTTGAGGAAGCGTCCGCCGAGTTGACCTCACAGAACATGCAGAAGGTCCAAGCTATTGGGTc                                                                                                                    <  1:5734/299‑1 (MQ=255)`  
                                                                                                                                                                                      `aTGAAGAGTACACACGAGATGGTCAATTTCAGTTATTTACAATCACGAAAGGAGGATAACCATATGTGTTGGGCAGCCGCAATACCTATCGCTATATCTGGCGCTCAGGCTATCAGTGATCAGAACGCTCAGGCCAAAATGATTGCCGCTCAGACCGCTGCTGGTCGTCGTCAAGCTATGGAAATCATGAGGCAGACGAACATCCAGAATGCTGACCTATCGTTGCAAGCTCGAAGTAAACTTGAGGAAGCGTCCGCCGAGTTGACCTCACAGAACATGCAGAAGGTCCAAGCTATTGGGt                                                                                                                     <  1:407047/301‑1 (MQ=255)`  
                                                                                                                                                                                       `agaagaaTACACACGAGATGGTCAATTTCAGTTATTTACAATCACGAAAGGAGGATAACCATATGTGTTGGGCAGCAGCAATACCTATCGCTATATCTGGCGCACAGGCTATCAGTGATCAGAACGCTCAGGCCAAAATGATTGCCGCTCAGACCGCTGCTGGTCGTCGTCAAGCTATGGAAATCATGAGGCAGACGAACATCCAGAATGCTGACCTATCGTTGCAAGCTCGAAGTAAACTTGAGGAAGCGTCCGCCGAGTTGACCTCACAGAACATGCAGAAGGTCCAAGCTATTGGGTc                                                                                                                    <  1:809672/300‑1 (MQ=255)`  
                                                                                                                                                                                        `gAAGAGTACACACGAGATGGTCAATTTCAGTTATTTACAATCACGAAAGGAGGATAACCATATGTGTTGGGCAGCCGCAATACCTATCGCTATATCTGGCGCTCAGGCTATCAGTGATCAGAACGCTCAGGCCAAAATGATTGCCGCTCAGACCGCTGCTGGTCGTCGTCAAGCTATGGAAATCATGAGGCAGACGAACATCCAGAATGCTGACCTATCGTTGCAAGCTCGAAGTAAACTTGAGGAAGCGTCCGCCGAGTTGACCTCACAGAACATGCAGAAGGTCCAAGCTATTGGGTCt                                                                                                                   >  1:641546/1‑301 (MQ=255)`  
                                                                                                                                                                                        `gAAGAGTACACACGAGATGGTCAATTTCAGTTATTTACAATCACGAAAGGAGGATAACCATATGTGTTGGGCAGCCGCAATACCTATCGCTATATCTGGCGCTCAGGCTATCAGTGATCAGAACGCTCAGGCCAAAATGATTGCCGCTCAGACCGCTGCTGGTCGTCGTCAAGCTATGGAAATCATGAGGCAGACGAACATCCAGAATGCTAACCTATCGTTGCAAGCTCGAAGTAAACTTGAGGAAGCGTCCGCCGAGTTGACCTCACAGAACATGCAGAAGGTCCAAGCTATTGGGTCt                                                                                                                   >  1:5438/1‑301 (MQ=255)`  
                                                                                                                                                                                           `gagTACACACGAGATGGTCAATTTCAGTTATTTACAATCACGAAAGGAGGATAACCATATGTGTTGGGCAGCCGCAATACCTATCGCTATATCTGGCGCTCAGGCTATCAGTGATCAGAACGCTCAGGCCAAAATGATTGCCGCTCAGACCGCTGCTGGTCGTCGTCAAGCTATGGAAATCATGAGGCAGACGAACATCCAGAATGCTGACCTATCGTTGCAAGCTCGAAGTAAACTTGAGGAAGCGTCCGCCGAGTTGACCTCACAGAACATGCAGAAGGTCCAAGCTATTGGGTCTATc                                                                                                                <  1:92288/301‑1 (MQ=255)`  
                                                                                                                                                                                           `gagTACACACGAGATGGTCAATTTCAGTTATTTACAATCACGAAAGGAGGATAACCATATGTGTTGGGCAGCCGCAATACCTATCGCTATATCTGGCGCTCAGGCTATCAGTGATCAGAACGCTCAGGCCAAAATGATTGCCGCTCAGACCGCTGCTGGTCGTCGTCAAGCTATGGAAATCATGAGGCAGACGAACATCCAGAATGCTGACCTATCGTTGCAAGCTCGAAGTAAACTTGAGGAAGCGTCCGCCGAGTTGACCTCACAGAACATGCAGAAGGTCCAAGCTATTGGGTCTATc                                                                                                                <  1:121044/301‑1 (MQ=255)`  
                                                                                                                                                                                             `gTACACACGAGATGGTCAATTTCAGTTATTTACAATCACGAAAGGAGGATAACCATATGTGTTGGGCAGCCGCAATACCTATCGCTATATCTGGCGCTCAGGCTATCAGTGATCAGAACGCTCAGGCCAAAATGATTGCCTCTCCGACCGCTGCTGGTCGTCGTCAAGCTATGGAAATCATGAGGCAGACGAACATCCAGAATGCTGACCTATCGTTGCAAGCCCGAAGTAAACTTGAGGAAGCGTCATCTGAGTTGACCTCACAGAACATGCAGAAGGTCCAGGATATTGGGTCTATccc                                                                                                              >  1:75859/1‑300 (MQ=255)`  
                                                                                                                                                                                             `gTACACACGAGATGGTCAATTTCAGTTATTTACAATCACGAAAGGAGGATAACCATATGTGTTGGGCAGCCGCAATACCTATCGCTATATCTGGCGCTCAGGCTATCAGTGATCAGAACGCTCAGGCCAAAATGATTGCCGCTCAGACCGCTGCTGGTCGTCGTCAAGCTATGGAAATCATGAGGCAGACGAACATCCAGAATGCTGACCTATCGTTGCAAGCTCGAAGTAAACTTGAGGAAGCGTCCGCCGAGTTGACCTCACAGAACATGCAGAAGGTCCAAGCTATTGGGTCTATCCg                                                                                                              >  1:619057/1‑301 (MQ=255)`  
                                                                                                                                                                                             `gTACACACGAGATGGTCAATTTCAGTTATTTACAATCACGAAAGGAGGATAACCATATGTGTTGGGCAGCCGCAATACCTATCGCTATATCTGGCGCTCAGGCTATCAGTGATCAGAACGCTCAGGCCAAAATGATTGCCGCTCAGACCGCTGCTGGTCGTCGTCAAGCTATGGAAATCATGAGGCAGACGAACATCCAGAATGCTGACCTATCGTTGCAAGCTCGAAGTAAACTTGAGGAAGCGTCCGCCGAGTTGACCTCACAGAACATGCAGAAGGTCCAAGCTATTGGGTCTATCCg                                                                                                              <  1:225716/301‑1 (MQ=255)`  
                                                                                                                                                                                             `gTACACACGAGATGGTCAATTTCAGTTATTTACAATCACGAAAGGAGGATAACCATATGTGTTGGGCAGCCGCAATACCTATCGCTATATCTGGCGCTCAGGCTATCAGTGATCAGAACGCTCAGGCCAAAATGATTGCCGCTCAGACCGCTGCTGGTCGTCGTCAAGCTATGGAAATCATGAGGCAGACGAACATCCAGAATGCTGACCTATCGTTGCAAGCGCGAAGTAAACTTGAGGAAGCGTCCGCCGAGTTGACCTCACAGACCATGCAGAAGGTCCAAGCTATTGGGTAGATCCg                                                                                                              >  1:306490/1‑301 (MQ=255)`  
                                                                                                                                                                                                     `gagaTGGTCAATTTCAGTTATTTACAATCACGAAAGGAGGATAACCATATGTGTTGGGCAGCCGCAATACCTATCGCTATATCTGGCGCTCAGGCTATCAGTGATCAGAACGCTCAGGCCAAAATGATTGCCGCTCAGACCGCTGCTGGTCGTCGTCAAGCTATGGAAATCATGAGGCAGACGAACATCCAGAATGCTGACCTATCGTTGCAAGCTCGAAGTAAACTTGAGGAAGAGTCCGCCGAGTTGACCTCACAGAACATGCAGAAGGTCCAAGCTATTGGGTCTATCCGATCGGCtt                                                                                                      >  1:52007/1‑300 (MQ=255)`  
                                                                                                                                                                                                           `ggcAATTTCAGTTATTTACAATCACGAAAGGAGGATAACCATATGTGTTGGGCAGCCGCAATACCTATCGCTATATCTGGCGCTCAGGCTATCAGTGATCAGAACGCTCAGGCCAAAATGATTGCCGCTCAGACCGCTGCTGGTCGTCGTCAAGCTATGGAAATCATGAGGCAGACGAACATCCAGAATGCTGACCTATCGTTGCAAGCTCGAAGTAAACTTGAGGAAGCGTCCGCCGAGTTGACCTCACAGAACATGCACAAGGTCCAAGCTATTGGGTCTATCCGAGCGGCTATCGgag                                                                                                <  1:368505/299‑1 (MQ=255)`  
                                                                                                                                                                                                             `agaattCAGTTAATTACAATCACGAAAGGATGATCACCATATGTGTTGGGCAGCAGCAATACATATCGCCATATCTGGCGCTCAGGCCATCAGTGATCAGAACGCTCAGGCCAAAATGATTGCCGCTCAGAGCGCTGCTGGTCGTCGTCAAGCTATGGAAATCATGAGGCAGACGCACATCCAGAATGCTGACCGATCGTTGCAAGCTCGCAGTACACTTGAGGAAGCGTCTGCCGAGGTGACCTCACAGAAAATGCAGAAGGTCCAAGCTATTGGGTCGATCAGAGCGGCTATCGgagag                                                                                              <  1:349842/297‑1 (MQ=255)`  
                                                                                                                                                                                                                     `aataagagacagcACGAAAGGAGGATAACCATATGTGTTGGGCAGCCGCAATACCTATCGCTATATCTGGCGCTCAGGCTATCAGTGATCAGAATGCTCAGGCCAAAATGATTGCCGCTCAGACCGCTGCTGGTCGTCGTCAAGCTATGGAAATCATGAGGCAGACGAACATCCAGAATGCTGACCTATCGTTGCAAGCTCGAAGTAAACTTGAGGAAGCGTCCGCCGAGTTGACCTCACAGAACATGCAGAAGGTCCAAGCTATTGGGTCTATCCGAGCGGCTATCGGAGAGAGTATGCt                                                                                      <  1:797207/289‑1 (MQ=255)`  
                                                                                                                                                                                                                           `tgtataagagacaggaggATAACCATATGTGTTGGGCAGCCGCAATACCTATCGCTATATCTGGCGCTCAGGCTATCAGTGATCAGAACGCTCAGGCCAAAATGATTGCCGCTCAGACCGCTGCTGGTCGTCGTCAAGCTATGGAAATCATGAGGCAGACGAACATCCAGAATGCTGACCTATCGTTGCAAGCTCGAAGTAAACTTGAGGAAGCGTCCGCCGAGTTTACCTCACAGAACATGCAGAAGGTCCAAGCTATTGGGTCTATCCGAGCGGCTATCGGAGAGAGTATGCTTGAAgg                                                                                <  1:627733/289‑1 (MQ=255)`  
                                                                                                                                                                                                                            `aCAATCACGAAAGGAGGATAACCATATGTGTTGGGCAGCCGCAATACCTATCGCTATATCTGGCGCTCAGGCTATCAGTGATCAGAACGCTCAGGCCAAAATGATTGCCTCTCAGACCGCTGCTGGTCGTCGTCAAGCTATGGAAATCATGAGGCAGACGAACATCCAGAATGCTGACCTATCGTTGCAAGCTCGAAGTAAACTTGAGGAAGCGTCCGCCGAGTTGACCTCACAGAACATGCAGAAGGTCCAAGCTATTGGGTCTATCCGAGCGGCTATCGGAGAGAGTATGCTTGAAGGt                                                                               <  1:596484/301‑1 (MQ=255)`  
                                                                                                                                                                                                                                   `atgtgtataagagacagtatGTGTTGGGCAGCCGCAATACCTATCGCTATATCTGGCGCTCAGGCTATCAGTGATCAGAACGCTCAGGCCAAAATGATTGCCGCTCAGACCGCTGCTGGTCGTCGTCAAGCTATGGAAATCATGAGGCAGACGAACATCCAGAATGCTGACCTATCGTTGCAAGCTCGAAGTAAACTTGAGGAAGCGTCCGCCGAGTTGACCTCACAGAACATGCAGAAGGTCCAAGCTATTGGGTATATCCGAGCGGCTATCGGAGAGAGTATGCTTGAAGGTTCCTCaa                                                                        <  1:734161/284‑1 (MQ=255)`  
                                                                                                                                                                                                                                         `gaggaTAATCATATGTGTTTGGCAGCCGCAATACCTATCGCTATATCTGGCGCTCAGGCTATCAGTGATCAGAACGCTCAGGCCAAAATGATTGCCGCTCAGACCGCTGCTGGTCGTCGTCAAGCTATGGAAATCATGAGGCAGACGAACATCCAGAATGCTGACCTATCGTTGCAAGCTCGAAGTAAACTTGAGGAAGCGTCCGCCGAGTTGACCTCACAGAACATGCAGAAGGTCCAAGCTATTGGGTCTATCCGAGCGGCTATCGGAGAGAGGATGCtctgtctcttatacacatctc                                                                  >  1:648522/1‑281 (MQ=255)`  
                                                                                                                                                                                                                                         `gaggaTAACCATATGTGTTGGGCAGCCGCAATACCTATCGCTATATCTGGCGCTCAGGCTATCAGTGATCAGAACGCTCAGGCCAAAATGATTGCCGCTCAGACCGCTGCTGGTCGTCGTCAAGCTATGGAAATCATAAGGCAGACGAACATCCAGAATGCTGACCTATCGTTGCAAGCTCGAAGTAAACTTGAGGAAGCGTCCGCCGAGTTGACCTCACAGAAAATGCAGAAGGTCCAAGCTATTGGTTCTATCCGAGCGACTATCGGAGTACTTATGCTTGAAGGTTCCTCAATTGAcc                                                                  >  1:342543/1‑301 (MQ=255)`  
                                                                                                                                                                                                                                           `ggaTAACCATATGTGTTGGGCAGCAGCAATACCTATCGCTATATCTGGCGCTCAGGCTATCAGTGATCAGAACGCTCAGGCCAAAATGATTGCCACTCAGACCGCTGCTGGTCGTCGTCAAGCTATGGAAATCATGAGGCAGACGAACATCCAGAATGCTGACCTATCGTTGCAAGCTCGAAGTAAACTTGAGGAAGCGTCCGCCGAGTTGACCTCACAGAACATGCAGAAGGTCCAAGCTATTGGGTCTATCCGAGCGGCTATCGGAGAGAGTATGCTTGAAGGTTCCTCAATGGACCGc                                                                >  1:719962/1‑301 (MQ=255)`  
                                                                                                                                                                                                                                                `aCCATATGTGTTGGGCAGCCGCAATACCTATCGCTATATCTGGCGCTCAGGCTATCAGTGATCAGAACGCTCAGGCCAAAATGATTGCCGCTCAGACCGCTGCTGGTCGTCGTCAAGCTATGGAAATCATGAGGCAGACGAACATCCAGAATGCTGACCGATCGTTGCAAGCTCGAAGTAAACTTGAGGAAGCGTCCGCCGAGTTGACCTAACAGAACATGCAGAAGGTCCAAGCTATTGGGTCTATCCGAGCGGCTATCGGAGAGAGTATGCTTGAAGGTTCCTCAATGGACCGCATTaa                                                           >  1:195034/1‑301 (MQ=255)`  
                                                                                                                                                                                                                                                 `ccATATGTGTTGGGCAGCCGCAATACCTATCGCTATATCTGGCGCTCAGGCTATCAGTGATCAGAACGCTCAGGCCAAAATGATTGCCGCTCAGACCGCTGCTGGTCGTCGTCAAGCTATGGAAATCATGAGGCAGACGAACATCCAGAATGCTGACCTATCGTTGCAAGCTCGAAGTAAACTTGAGGAAGCGTCCGCCGAGTTGACCTCACAGAACATGCAGAAGGTCCAAGCTATTGGGTCTATCCGAGCGGCTATCGGAGAGAGTATGCTTGAAGGTTCCTCAATGGACCGCATTAAg                                                          >  1:68178/1‑301 (MQ=255)`  
                                                                                                                                                                                                                                                   `gggctcggagatgtgtataagagacagtCGCTATATCTGGCGCTCAGGCTATCAGTGATCAGAACGCTCAGGCCAAAATGATTGCCGCTCAGACCGCTGCTGGTCGTCGTCAAGCTATGGAAATCATGAGGCAGACGAACATCCAGAATGCTGACCTATCGTTGCAAGCTCGAAGTAAACTTGAGGAAGCGTCCGCCGAGTTGACCTCACAGAACATGCAGAAGGTCCAAGCTATTGGGTCTATCCGAGCGGCTATCGGAGAGAGTATGCTTGAAGGTTCCTCAATGGACCGCATTAAGCg                                                        <  1:270248/274‑1 (MQ=255)`  
                                                                                                                                                                                                                                                   `atatGTGTTGGGCAGCCGCAATACCTATCGCTATATCTGGCGCTCATGCTATCAGTGATCAGAACGCTCAGGCCAAAATGATTGCCGCTCAGGCCGCTGCTGGTCGTCGTCAAGCTATGGAAATCATGAGGCAGACGAACATCCAGAATGCTGACCTATCGTTGCAAGCTCGAAGTAAACTTGAGAAAGCGTCCGCCGAGTTGACCTCACAGAACATGCAGAAGGTCCAAGCTATTGGGTCTATCCGAGCGGCTATCGGAGAGAGTATGCTTGAAGGTTCCTCAATGGACCGCATTAAGCg                                                        <  1:384983/301‑1 (MQ=255)`  
                                                                                                                                                                                                                                                    `tatGTGTTGGGCAGCCGCAATACCTATCGCTATATCTGGCGCTCAGGCTATCAGTGATCAGAACGCTCAGGCCAAAATGATTGCCGCTCAGACCGCTGCTGGTCGTCGTCAAGCTATGGAAATCATGAGGCAGACGAACATCCAGAATGCTGACCTATCGTTGCAAGCTCGAAGTAAACTTGAGGAAGCGTCCGCCGAGTTGACCTCACAGAACATGCAGAAGGTCCAAGCTATTGGGTCTATCCGAGCGGCTATCGGAGAGAGTATGCTTGAAGGTTCCTCAATGGACCGCATTAAGCGa                                                       >  1:79071/1‑301 (MQ=255)`  
                                                                                                                                                                                                                                                       `gtgtTGGTCAGCCGCAATACCTATCGCTATATCTGGCGCTCAGGCTATCAGTGATCAGAACGCTCAGGCCAAAATGATTGCCGCTCAGACCGCTGCTGGTCGTCGTCAAGCTATGGAAATCATGAGGCAGACGAACATCCAGAATGCTGACCTATCGTTGCAAGCTCGAAGTAAACTTGAGGAAGCGTCCGCCGAGTTGACCTCACAGAACATGCAGAAGGTCCAAGCTATTGGGTCTATCCGAGCGGCTATCGGAGAGAGTATGCTTGAAGGTTCCTCAATGGACCGCATTAAGCGAGTc                                                    >  1:298433/1‑301 (MQ=255)`  
                                                                                                                                                                                                                                                       `gtgtTGGGCAGCCGCAATACCTATCGCTATATCTGGCGCTCAGGCTATCAGTGATCAGAACGCTCAGGCCAAAATGATTGCCGCTCAGACCGCTGCTGGTCGTCGTCAAGCTATGGAAATCATGAGGCAGACGAACATCCAGAATGCTGACCTATCGTTGCAAGCTCGAAGTAAACTTGAGGAAGCGTCCGCCGAGTTGACCTCACAGAACATGCAGAAGGTCCAAGCTATTGGGTCTATCCGAGCGGCTATCGGAGAGAGTATGCTTGAAGGTTCCTCAATGGACCGCATTAAGCGAGta                                                    >  1:142582/1‑300 (MQ=255)`  
                                                                                                                                                                                                                                                                      `aaTACCTATCGCTATATCTGGCGCTCAGGCTAGCAGTGATCAGAACGCTCAGGCCAAAATGATTGCCGCTCAGACCGCTGCTGGTCGTCGTCAAGCTATGGAAATCATGAGGCAGACGAACATCCAGAATGCTGACCTATCGTTGCAAGCTCGAAGTAAACTTGAGGAAGCGTCAGCCGAGTTGACCTCACAGAACATGCAGAAGGTCCAAGCTATTGGGTCTATCCGAGCGGCTATCGGAGAGAGTATGCTTGAAGGTTCCTCAATGGACCGCATTAAGCGAGTCACAGAAGGACAGTTc                                     <  1:392421/301‑1 (MQ=255)`  
                                                                                                                                                                                                                                                                      `aaTACCTATCGCTATATCTGGAGCTCAGGCTATCAGTGATCAGAACGCTCAGGCCAAAATGATTGCCGCTCAGACCGCTGCTGGTCGTCGTCAAGCTATGGAAATCATGAGGCAGACGAACATCCAGAATGCTGACCTATCGTTGCAAGCTCGAAGTAAACTTGAGGAAGCGTCCGCCGAGTTGACCTCACAGAACATGCAGAAGGTCCAAGCTATTGGGTCTATCCGAGCGGCTATCGGAGAGAGTATGCTTGAAGGTTCCTCAATGGACCGCATTAAGCGAGTCACAGAAGGACAGTTc                                     <  1:741983/301‑1 (MQ=255)`  
                                                                                                                                                                                                                                                                         `aCCTATCGCTATATCTGGCGCTCAGGCTATCAGTGATCAGAACGCTCAGGCCAAAATGATTGCCGCTCAGACCGCTGCTGGTCGTCGTCAAGCTATGGAAATCATGAGGCAGACGAACATCCAGAATGCTGACCTATCGTTGCAAGCTCGAAGTAAACTTGAGGACGCGTCCGCCGAGTTGACCTCACAGAACATGCAGAAGGTCCAAGCTATTGGGTCTATCCGAGCGGCTATCGGAGAGAGTATGCTTGAAGGTTCCTCAATGGACCGCATTAAGCGAGTCACAGAAGGACAGTTCCtt                                  >  1:185114/1‑301 (MQ=255)`  
                                                                                                                                                                                                                                                                                   `atatCTGGCGCTCAGGCTATCAGTGATCAGAACGCTCAGGCCAAAATGATTGCCGCTCAGACCGCTGCTGGTCGTCGTCAAGCTATGGAAATCATGAGGCAGACGAACATCCAGAATGCTGACCTATCGTTGCAAGCTCGAAGTAAACTTGAGGAAGCGTCCGCCGAGTTGACCTCACAGAACATGCAGAAGGTCCAAGCTATTGGGTCTATCCGAGCGGCTATCGGAGAGAGTATGCTTGAAGGTTCCTCAATGGACCGCATTAAGCGAGTCACAGAAGGAcctgtctcttatacacatc                        >  1:587723/1‑283 (MQ=255)`  
                                                                                                                                                                                                                                                                                       `taagagacagggCTATCAGTGATCAGAACGCTCAGGCCAAAATGATTGCCGCTCAGACCGCTGCTGGTCGTCGTCAAGCTATGGAAATCATGAGGCAGACGAACATCCAGAATGCTGACCTATCGTTGCAAGCTCGAAGTAAACTTGAGGAAGCGTCCGCCGAGTTGACCTCACAGAACATGCAGAAGGTCCAAGCTATTGGGTCTATCCGAGCGGCTATCGGAGAGAGTATGCTTGAAGGTTCCTCAATGGACCGCATTAAGCGAGTCACAGAAGGACAGTTCATTCGGGAAGCCAatat                    <  1:400240/291‑1 (MQ=255)`  
                                                                                                                                                                                                                                                                                        `aagagacagggCTATCAGTGATCAGAACGCTCAGGCCAAAATGATTGCCGCTCAGACCGCTGCTGGTCGTCGTCAAGCTATGGAAATCATGAGGCAGACGAACATCCAGAATGCTGACCTATCGTTGCAAGCTCGAAGTAAACTTGAGGAAGCGTCCGCCGAGTTGACCTCACAGAACATGCAGAAGGTCCAAGCTATTGGGTCTATCCGAGCGGCTATCGGAGAGAGTATGCTTGAAGGTTCCTCAATGGACCGCATTAAGCGAGTCACAGAAGGACAGTTCATTCGGGAAGCCAATATg                   <  1:254697/292‑1 (MQ=255)`  
                                                                                                                                                                                                                                                                                         `ggCGCTCAGGCTATCAGTGATCAGAACGCTCAGGCCAAAATGATTGCCGCTCAGACCGCTGCTGGTCGTCGTCAAGCTATGGAAATCATGAGGCAGACGAACATCCAGAATGCTGACCTCTCGTTGCAAGCTCGAAGTAAACTTTAGGAAGCGTCCGCCGAGTTGACCTCACAGAACATGCAGAAGGTCCAAGCTATTGGGTCTATCCGAGCGGCTATCGGAGAGAGTATGCTTGAAGGTTCCTCAATGGACCGCATTAAGCGAGTCACAGAAGGACAGTTCATTCGGGAAGCCAATATgg                  >  1:177613/1‑301 (MQ=255)`  
                                                                                                                                                                                                                                                                                           `cgcTCAGGCTATCAGTGATCAGAACGCTCAGGCCAAAATGATTGCCGCTCAGACCGCTGCTGGTCGTCGTCAAGCTATGGAAATCATGAGGCAGACGAACATCCAGAATGCTGACCTATCGTTGCAAGCTCGAAGTAAACTTGAGGAAGCGTCCGCCGAGTTGACCTCACAGAACATGCAGAAGGTCCAAGCTATTGGGTCTATCCGAGCGGCTATCGGAGAGAGTATGCTTGAAGGTTCCTCAATGGACCGCATTAAGCGAGTCACAGAAGGACAGTTCATTCGGGAAGCCAatatctgt                >  1:691866/1‑297 (MQ=255)`  
                                                                                                                                                                                                                                                                                             `cTCAGGCTATCAGTGATCAGAACGCTCAGGCCAAAATGATTGCCGCTCAGACCGCTGCTGGTCGTCGTCAAGCTATGGAAATCATGAGGCAGACGAACATCCAGAATGCTGACCTATCGTTGCAAGCTCGAAGTAAACTTGAGGAAGCGTCCGCCGAGTTGACCTCACAGAACATGCAGAAGGTCCAAGCTATTGGGTCTATCCGAGCGGCTATCGGAGAGAGTATGCTTGAAGGTTCCTCAATGGACCGCATTAAGCGAGTCACAGAAGGACAGTTCATTCGGGAAGCCAATATGGTAAc              <  1:658803/301‑1 (MQ=255)`  
                                                                                                                                                                                                                                                                                             `acagggCTATCAGTGATCAGAACTCTCAGGCCAAAATGATTGCCGCTCAGACCGCTGCTGGTCGTCGTCAAGCTATGGAAATCATGAGGCAGACGAACATCCAGAATGCTGACCTATCGTTGCAAGCTCGAAGTAAACTTGAGGAAGCGTCCGCCGAGTTGACCTCACAGAACATGCAGAATGTCCAAGCTATTGGGTCTATCCGAGCGGCTATCGGAGAGAGTATGCTTGAAGGTTCCTCAATGGACCGCATTAAGCGAGTCACAGAAGGACAGTTCATTCGGGAAGCCAATATGGTAAc              <  1:767530/297‑1 (MQ=255)`  
                                                                                                                                                                                                                                                                                             `aagagacagtCAGTGATCAGAACGCTCAGGCCAAAATGATTGCCGCTCAGACCGCTGCTGGTCGTCGTCAAGCTATGGAAATCATGAGGCAGACGAACATCCAGAATGCTGACCTATCGTTGCAAGCTCGAAGTAAACTTGAGGAAGCGTCCGCCGAGTTGACCTCACAGAACATGCAGAAGGTCCAAGCTATTGGGTCTATCCGAGCGGCTATCGGAGAGAGTATGCTTGAAGGTTCCTCAATGGACCGCATTAAGCGAGTCACAGAAGGACAGTTCATTCGGGAAGCCAATATGGTAAc              <  1:706793/292‑1 (MQ=255)`  
                                                                                                                                                                                                                                                                                               `cAGGCTATCAGTGATCAGAACGCTCAGGCCAAAATGATTGCCGCTCAGACCGCTGCTGGTCGTCGTCAAGCTATGGAAATCATGAGGCAGACGAACATCCAGAATGCTGACCTATCGTTGCAAGCTCGAAGTAAACTTGAGGAAGCGTCCGCCGAGTTGACCTCACAGAACATGCAGAAGGTCCAATCTATTGGGTCTATCCGAGCGGCTATCGGAGAGAGTATGCTTGAAGGTTCCTCAATGGACCGCATTAATCGAGTCACAGAAGGACAGTTCATTCGGGAAGCCAATATGGTAACTg            <  1:581958/301‑1 (MQ=255)`  
                                                                                                                                                                                                                                                                                                      `tCAGTGATCAGAACGCTCAGGCCAAAATGATTGCCGCTCAGACCGCTGCTGGTCGTCGTCAAGCTATGGAAATCATGAGGCAGACGAACATCCAGAATGCTGACCTATCGTTGCAAGCTCGAAGTAAACTTGAGGAAGCGTCCGCCGAGTTGACCTCACAGAACATGCAGAAGGTCCAAGCTATTGGGTCTATCCGAGCGGCTATCGGAGAGAGTATGCTTGAAGGTTCCTCAATGGACCGCATTAAGCGAGTCACAGAAGGACAGTTCATTCGGGAAGCCAATATggctgtctcttatac     >  1:249183/1‑288 (MQ=255)`  

                                                                                                                                                                                                                                                                                                            `|`                                                                                                                                                                                                                                                                                                           
`GTGCTGGTTCGTTACGAGCGACCAAGTGTGGCGACTTAGTGGAAAGGCTAAGCGAAAGTTCCGTAAGTTAATCATGGAGTATCGCGATAAGATGCTTGAGAAGTATGATACTCTTTGGAATTACGTATGGGTAGGCAATACGTCCCACATTCGTTTCCTCAAGACTATCGGTGCGGTATTCCATGAAGAGTACACACGAGATGGTCAATTTCAGTTATTTACAATCACGAAAGGAGGATAACCATATGTGTTGGGCAGCCGCAATACCTATCGCTATATCTGGCGCTCAGGCTATCAGTGGTCAGAACGCTCAGGCCAAAATGATTGCCGCTCAGACCGCTGCTGGTCGTCGTCAAGCTATGGAAATCATGAGGCAGACGAACATCCAGAATGCTGACCTATCGTTGCAAGCTCGAAGTAAACTTGAGGAAGCGTCCGCCGAGTTGACCTCACAGAACATGCAGAAGGTCCAAGCTATTGGGTCTATCCGAGCGGCTATCGGAGAGAGTATGCTTGAAGGTTCCTCAATGGACCGCATTAAGCGAGTCACAGAAGGACAGTTCATTCGGGAAGCCAATATGGTAACTGAGAACTATCG  >  NC_001604.1/27483‑28080`

  

Alignment Legend

Aligned base mismatch/match (shaded by quality score): `ATCG/ATCG` `< 3 ≤` `ATCG/ATCG` `< 11 ≤` `ATCG/ATCG` `< 25 ≤` `ATCG/ATCG` `< 36 ≤` `ATCG/ATCG` `< 38 ≤` `ATCG/ATCG`

Unaligned base: `atcg`    Masked matching base: `atcg`    Alignment gap: `‑`    Deleted base: `‑`
